# Worksite Tobacco Prevention: A Randomized, Controlled Trial of Adoption, Dissemination Strategies, and Aggregated Health-Related Outcomes across Companies

**DOI:** 10.1155/2015/136505

**Published:** 2015-10-04

**Authors:** Verena Friedrich, Adrian Brügger, Georg F. Bauer

**Affiliations:** ^1^Centre of Continuing Education, University of Bern, Schanzeneckstrasse 1, 3001 Bern, Switzerland; ^2^Institute of Marketing and Management, Department of Consumer Behavior, University of Bern, 3012 Bern, Switzerland; ^3^Epidemiology, Biostatistics and Prevention Institute, University of Zurich, 8001 Zurich, Switzerland

## Abstract

Evidence based public health requires knowledge about successful dissemination of public health measures. This study analyses (a) the changes in worksite tobacco prevention (TP) in the Canton of Zurich, Switzerland, between 2007 and 2009; (b1) the results of a multistep versus a “brochure only” dissemination strategy; (b2) the results of a monothematic versus a comprehensive dissemination strategy that aim to get companies to adopt TP measures; and (c) whether worksite TP is associated with health-related outcomes. A longitudinal design with randomized control groups was applied. Data on worksite TP and health-related outcomes were gathered by a written questionnaire (baseline *n* = 1627; follow-up *n* = 1452) and analysed using descriptive statistics, nonparametric procedures, and ordinal regression models. TP measures at worksites improved slightly between 2007 and 2009. The multistep dissemination was superior to the “brochure only” condition. No significant differences between the monothematic and the comprehensive dissemination strategies were observed. However, improvements in TP measures at worksites were associated with improvements in health-related outcomes. Although dissemination was approached at a mass scale, little change in the advocated adoption of TP measures was observed, suggesting the need for even more aggressive outreach or an acceptance that these channels do not seem to be sufficiently effective.

## 1. Introduction

Measures for tobacco prevention (TP) at the worksite are a key strategy of tobacco control [1], since many people spend a great part of their day at work, where second-hand smoke (SHS) is still common. As Kramer et al. [2] showed in their synopsis of the literature, structural TP measures (especially smoke-free policies) and behavioural prevention measures (e.g., group interventions and consultations for smokers) at the worksite contribute to reducing the prevalence of smoking and cigarette consumption among staff [3], to improved air quality [4], and reduced absenteeism [5].

However, for these TP measures to have a large public health impact, they must be disseminated across organizations and adopted by them [6, 7]. Laws or regulations for the protection of nonsmokers, which have been introduced in many countries in recent years, have contributed significantly to the dissemination of smoke-free policies. However, as Radtke et al. [8] showed for Switzerland, 28% of the working population were still exposed to SHS at the worksite in 2010, even though the Swiss Federal law for protection against passive smoking was implemented in that year. It is therefore an ongoing challenge for public health professionals to sensitize decision-makers and support companies in the implementation of TP measures, both in countries with and without workplace smoking policies. Although several studies have addressed the dissemination of health promotion interventions associated with different health topics and settings [9], no study has yet addressed the dissemination of TP across worksites.

**Figure 1:**
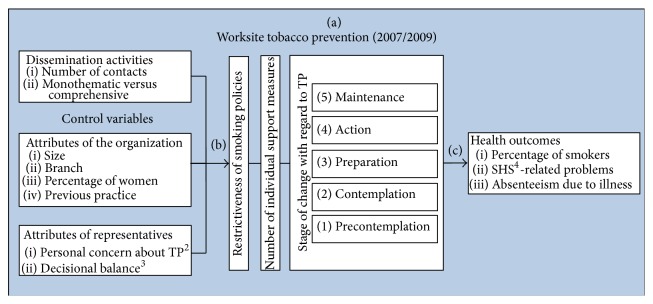
Variables to be analysed with regard to changes of worksite TP (research question a), predictors (question b), and outcomes (question c) of worksite TP.* Notes.* ^1^Other workplace health promotion measures; ^2^tobacco prevention; ^3^pros and cons regarding WHP; ^4^second-hand smoke.

Accordingly, this study aims to contribute to the knowledge of dissemination and adoption of TP across companies by addressing the following research questions (Figure 1). It presents follow-up data to a 2007 survey of more than 1400 companies in the Canton of Zurich, Switzerland (for the baseline results see [10]).

- In what way did the overall adoption of TP by companies in the Canton of Zurich, Switzerland, change between 2007 and 2009?
- Does the implementation of a multistep dissemination strategy to promote TP in companies produce larger effects regarding the adoption of TP than only sending out an information brochure to companies?
- Does embedding the topic of TP in a comprehensive workplace health promotion (WHP) dissemination strategy lead to better adoption of worksite TP than promoting TP in isolation (monothematic dissemination strategy)?
- Does improved worksite TP lead to improved health-related outcomes aggregated at company level?

(a) Firstly, regarding the overall adoption of worksite TP, we examine how its prevalence changed across companies between 2007 and 2009. In this period, the Swiss Federal law for protection against passive smoking was not yet in force, so companies were relatively free in their application of nonsmoking regulations. Worksite TP includes smoking restriction policies (spatial restrictions) and a number of individual support measures offered to smokers (e.g., cessation courses). Besides looking at the full adoption of these preventive measures by companies, we also use the transtheoretical model of change to analyse the five stages of change (SoC) of companies in the adoption process [11]: precontemplation, contemplation, preparation, action, and maintenance. This is a more differentiated, sensitive measure covering both attitudinal and behavioural aspects associated with future behaviour change and clearly distinguishes between short-term behaviour change (action) and long-term behaviour change (maintenance).

(b1) Secondly, we analyse if the implementation of a multistep dissemination strategy in companies to promote TP is more effective for the adoption of TP than only sending out an information brochure. A systematic review of dissemination studies concluded that more active, multimodal dissemination and implementation strategies are more likely to be effective [9]. In comparing the “brochure only” to the multistep strategy, we control for variables that are known to be associated with TP at worksites [10]: the characteristics of the organization (e.g., size, industry type, and previous WHP practice) and the characteristics of the decision-makers within the organization (personal concern for TP, perceived advantages of TP).

(b2) Thirdly, we examine whether an approach to dissemination presenting TP as an integral part of a comprehensive WHP strategy yields better results than an approach presenting TP in isolation. The rationale for comprehensive WHP has been widely discussed [12, 13]. Regarding worksite TP, the aim of integrating it into a broader WHP strategy is to discuss factors associated with smoking cessation (e.g., body weight and diet; assumed stress relief) or with the introduction of smoke-free worksites (e.g., corporate communications, team climate) in the more positively connoted context of promoting health at work. In comparing the comprehensive and monothematic dissemination strategies, we control for the above mentioned variables (characteristics of the organisation and the representative).

(c) Fourthly, we examine whether improved worksite TP is associated with improved health outcomes aggregated at the company level. We included outcomes reported to be positively affected by workplace TP: the percentage of smokers in the workforce [3], SHS-exposure and related complaints [4], and the absenteeism rate due to illness [5].

## 2. Material and Methods

### 2.1. Study Population and Study Design

**Figure 2:**
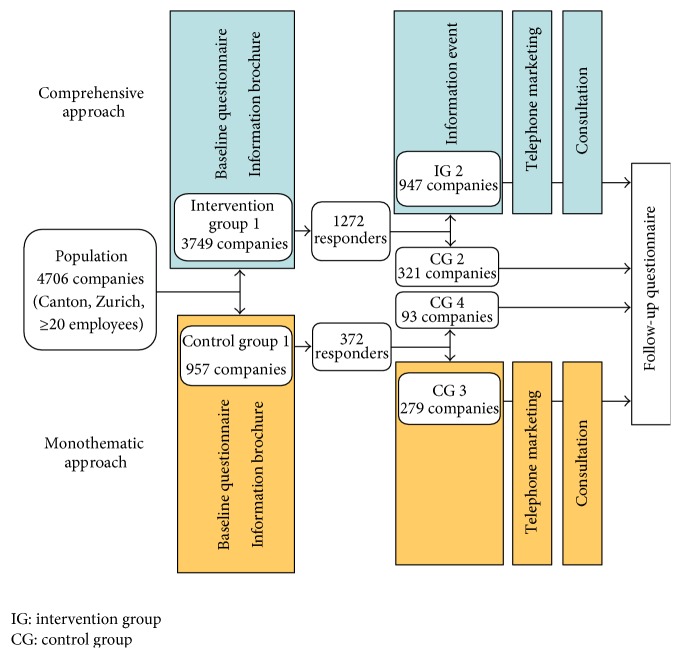
Intervention and study design.

The study included all companies in the Canton of Zurich, Switzerland, with 20 or more employees (*N* = 4706), varying in their baseline levels of worksite TP (see Table 1). This study population was randomly divided into intervention and control groups (simple random allocation using SPSS random number allocator; see Figure 2). Intervention group 1 received a “comprehensive” dissemination strategy in which TP was part of a comprehensive approach to WHP. Control group 1 received a “monothematic” dissemination strategy with TP as a singular topic. The comprehensive dissemination strategy was carried out by Zurich University's WHP consulting team. The monothematic dissemination strategy was implemented by TP specialists from the Swiss Lung League, a national organization for the prevention and control of lung diseases.

In a first step, both intervention group 1 and control group 1 received bulk mailings with information brochures and the baseline questionnaire. The “comprehensive” brochure explained the benefits of worksite TP and WHP and of their integration, showed how to proceed in practice, and gave information about the consulting agency's offers. The “monothematic” brochure provided information about the benefits of worksite TP, about related support services, and invited companies to register on a website as smoke-free workplaces. As the subsequent dissemination activities could not be offered to all companies due to limited resources, the companies who had answered the baseline questionnaire were further randomly subdivided into intervention group 2 (IG 2; comprehensive intervention), control group 3 (CG 3; monothematic intervention), and control groups 2 and 4 (CG 2 and 4, no further intervention).

Next, intervention group 2 companies were invited to information events. As only 29 of 947 companies participated, this step was omitted for control group 3. The subsequent steps were telephone marketing (selectively calling companies that had expressed interest in WHP services in the baseline questionnaire; intervention group 2: 133 companies reached; control group 3: 80 companies reached) and free initial consultations (intervention group 2: 92 companies; control group 3: 49 companies. These numbers were a subsample of those reached by the previous step). The dissemination activities are described in detail in [14].

### 2.2. Data Collection

Company addresses and information about company size (number of employees) and branch were obtained from the Federal Statistics Office. Data on dissemination activities implemented by the dissemination teams across companies were recorded in a customer database. The following measures of dissemination activities were derived: presence or absence of any dissemination activity apart from the brochure (1/0); number of contacts between dissemination team and company; and type of dissemination activity (0 = brochure only, 1 = monothematic intervention, and 2 = comprehensive intervention). The other variables (Figure 1) were measured using a written questionnaire addressed to the human resources or occupational health managers as representatives of the companies who should have the best overview of the relevant variables. The baseline measurement was made in June 2007 (*t*1); a follow-up measurement was made in March 2009 after the dissemination activities had been implemented (*t*2). Both questionnaires were sent to all companies in the Canton of Zurich, Switzerland, with 20 or more employees (*t*1  *N* = 4706; *t*2  *N* = 4472). The questionnaire was the same for the baseline and follow-up levels. Details of the variables (number of items, response format) are described in the Appendix and in [10].

### 2.3. Participating Sample

Of the questionnaires sent to 4706 companies at baseline level, 1648 were returned (response rate after excluding undeliverable questionnaires: 36.5%). Of those, 1627 could be analyzed. For the follow-up, undeliverables were excluded from the addressees, resulting in a population of *N* = 4472. A total of 1502 questionnaires were completed at follow-up, of which 1452 were suitable for analyses. A total of 244 questionnaires were returned blank because the company had fewer than 20 employees, moved away, or had ceased to exist. After subtracting these from the population, the response rate was 35.5%. A total of 827 companies returned both the baseline and follow-up questionnaires. Compared to data from the Federal Statistics Office, the survey samples are representative of worksites in the Canton of Zurich as regards company size and the four major branches (construction, hospitality, healthcare/welfare, and trading/maintenance/repair), except that healthcare and welfare organizations are slightly overrepresented. Regarding the characteristics of the organizations and representatives, the follow-up sample is comparable to the baseline sample (see Appendix and [10]). At follow-up, 64% of the participating companies had 20–49 employees, 30% 50–250 employees, and 5% more than 250 employees at baseline. Of the representatives who completed the questionnaire at follow-up, 72% were nonsmokers, 48% were CEOs, and 44% were human resource managers. Irrespective of their position, 49% reported that they were authorized to decide on occupational health measures in their companies.

### 2.4. Data Analyses

Descriptive statistics and Wilcoxon tests were used to examine the changes in worksite TP (policy restrictiveness, number of individual support measures) and stages of change regarding smoke-free policy and cessation course between 2007 and 2009 (research question a).

To answer research questions (b1) and (b2),* t*-tests and ordinal regression analyses were performed, with control variables (attributes of the organization and the representatives; measured at* t*1) and dissemination strategies as factors ((b1) brochure only versus brochure and other, number of contacts; (b2) monothematic versus comprehensive strategy), and differences in worksite TP (policy restrictiveness, individual support measures) and stages of change (smoke-free policy, cessation course) between 2009 and 2007 (*t*2 minus* t*1 data) as dependent variables.

Ordinal regression analyses were performed to test whether changes in TP (policy restrictiveness, individual support measures) and the organizations' stage of change (smoke-free policy, cessation course) predict improvements in health-related outcomes (percent of smokers, second-hand smoke related problems, and absenteeism; research question c). We used categorized changes in TP (−1 = decrease, 0 = no change, and 1 = increase) as factors and differences (*t*2 minus* t*1 data) in health-related outcomes as dependent variables.

## 3. Results

### 3.1. Changes in Adoption of Worksite TP between 2007 and 2009

A comparison of cross-sectional data in 2007 and 2009 showed that the proportion of organizations with no smoking regulations (or smoking allowed anywhere) was slightly reduced (see Table 1). The largest change was found in tightening the policy from “smoking is allowed outside and in certain indoors areas” to “allowed outside, not in buildings.” The number of individual support measures (e.g., cessation courses) did not differ between the two years. In 2009, more companies were in a higher stage of change (SOC) concerning the introduction of smoke-free policies, with a particularly large increase of the maintenance stage. The SOC regarding cessation courses remained low.

Longitudinal analyses showed that the changes within companies participating in the baseline and follow-up surveys (*n* = 827) were also rather small, but conformed to the cross-sectional data. Wilcoxon tests analysed whether changes between* t*2 and* t*1 data in worksite TP (−1 = decrease, 0 = no change, and 1 = increase) were statistically significant (Table 2). The results indicated that more companies tightened their smoking policies than those adopting a looser policy. There were also more companies with an SOC increase in smoke-free policy and cessation courses than those with an SOC decrease. However, effect sizes for these differences were rather small [15].

### 3.2. Effect of Dissemination Strategies on the Adoption of Worksite TP

In a first step,* t*-tests assessed whether control groups 2 (comprehensive brochure only) and 4 (monothematic brochure only) differed with regard to changes in worksite TP (*t*2 minus* t*1 data). As shown in Table 3, there were no significant differences between the two groups. Therefore, CG 2 and CG 4 data were pooled for the following analyses.

To analyse the relationship between control variables, interventions, and the adoption of worksite TP, we performed ordinal regression analyses (Table 4). The results showed that construction companies improved more than companies in other branches with regard to the restrictiveness of their TP policy, and hospitality venues improved less. However, construction companies improved less with regard to the number of individual support measures. Health and welfare organizations improved more in their stage of change regarding cessation courses than other sectors. Companies that had adopted many other WHP measures at* t*1 improved less in their SoC regarding a smoke-free policy. The same is true for companies that reported high personal concern and perceived advantages at* t*1.

As to the effect of the dissemination strategies, the results showed that the “brochure only” intervention (control groups 2 and 4) was associated with less improvement in individual support measures than any additional (monothematic or comprehensive) interventions. However, the number of additional contacts were not predictive of the outcomes. In comparing the dissemination strategies, we found no significant differences between intervention group 2 (comprehensive dissemination strategy) and control group 3 (monothematic dissemination strategy). However, the results at least show a statistically not significant tendency for the comprehensive intervention to be superior as regards policy restrictiveness and the related SoC, whereas the monothematic intervention was superior as regards outcomes relating to smoking cessation.

### 3.3. Effect of Worksite TP on Health-Related Outcomes

In the entire sample, more companies reported an increased percentage of smokers in their workforce than a decreased one (Table 2), whereas problems related to environmental tobacco smoke decreased. Absenteeism due to illness did not change significantly. As regards the question of whether changes in worksite TP predict changes in health-related outcomes, ordinal regression analyses with categorized changes in TP as factors showed that less restrictive policies and a decreased SoC regarding smoke-free policies were associated with an increased percentage of smokers in the workforce and increased SHS-related problems between 2007 and 2009 (Table 5). A decreased SoC regarding smoke-free policies is also associated with more absenteeism due to illness. Decreased and unchanged numbers of individual support measures are associated with more SHS-related problems, whereas a decreased SoC regarding cessation courses is associated with a higher percentage of smokers in the workforce.

## 4. Discussion

This study shows that in 2009 only half of the worksites in the Canton of Zurich, Switzerland, had adopted an effective smoking policy (48% banned smoking indoors, 2.3% anywhere). Only few offered individual support measures for smokers. Longitudinal analysis (research question a) showed slight but significant improvements in worksite TP, notably regarding smoking policies. Companies in the construction sector improved more than other companies, probably because they started from a considerably lower level of TP in 2007. In contrast, hospitality venues improved less. Protection from SHS for employees in hospitality venues still remains inconsistent and suboptimal, as the Swiss Federal law for protection against passive smoking from 2010 still allows exemptions from the smoking ban in this sector [16].

The other control variables (previous practice, personal concern, and perceived advantages) that predicted the state of worksite TP at baseline (cf. [10]) are not or negatively associated with improvements in worksite TP, measured as* t*2 minus* t*1 data. This may be due to the fact that companies with high levels of these control variables already had higher levels of TP at* t*1 [10] and thus less room for improvement.

Regarding research question (b1), the results showed that, compared to the “brochure only” groups, additional (monothematic or comprehensive) dissemination activities had a positive impact on the number of individual support measures and the related SoC of companies. However, this effect could not be found for the outcomes relating to smoking policy. This can be explained by the societal context of this study: it took place during the run-up to the Federal law for protection against passive smoking at worksites, which was accompanied by vigorous public debates over the benefit of smoke-free policies, and as part of a general trend towards such policies (e.g., a nonsmoking policy in Swiss trains had been introduced in 2005). Thus, the dissemination activities probably could not generate any effect on worksite smoking policies in* addition* to the historical developments that were strong enough to produce significant results in the longitudinal analysis. Also, the number of contacts between the intervention teams and the companies (in addition to brochure) did not predict improvement in any of the worksite TP measures. Research question (b2) focused on a comparison of the monothematic and comprehensive dissemination strategy. Although the data (bottom of Table 3) suggest that, compared to the monothematic dissemination strategy, the comprehensive strategy led to greater improvements in policy restrictiveness, whereas the monothematic dissemination strategy was superior as regards the improvement of individual support measures, no statistically significant differences between the two dissemination strategies were found.

With respect to research question (c), this study showed that changes in worksite TP are associated with changes in relevant health-related outcomes aggregated on the company level. In particular, a less restrictive policy, less individual support measures, and a decreased stage of change regarding smoke-free policy predict an increase in SHS-related problems. That is, maintaining positive changes in organizations seems to pay off. However, these findings should be interpreted with caution as they rely on self-reporting from a single representative within an organization.

The strengths of the study lie in its longitudinal, randomized controlled trial design, the large sample size, and the real-world setting in which the interventions were studied, as urged by Rabin et al. [9]. The heterogeneity of companies included in this broad field study assures that the results are generalizable to contexts where legal regulations are debated but not yet in force.

However, this field setting also caused some limitations: first, the compared dissemination strategies differed not only with respect to the implementation teams but also with respect to the steps taken: the information events in the intervention group were omitted for the control groups. Second, for feasibility reasons, baseline questionnaires and information brochures were sent in the same mailing; to achieve a sound baseline, the questionnaires should have been sent* before* the brochures, also allowing for a no-brochure control group. Third, self-selection in survey participation in this study probably led to an overestimation of the actual prevalence of TP, since more advanced companies are more likely to participate. Also, the companies that participated in the information events and in initial consultations presumably attach greater importance to the wellbeing of their employees and have the resources to invest in TP or broader WHP activities. This does not limit the external validity of the results, as this selection bias will also apply to future efforts to disseminate voluntary TP measures outside a study context. However, this is problematic from a public health equity perspective, because less privileged groups of employees remain disadvantaged and have less chance of benefiting from voluntary TP and WHP. Thus, the importance of consistent legal regulations regarding tobacco control reaching all companies independently of existing resources and motivation for TP becomes even clearer.

## 5. Conclusions

This is the first large-scale, longitudinal field study examining the active dissemination and adoption of TP across companies from all economic sectors in a region. Over a period of two years (2007–2009), it showed slight improvements in worksite TP, specifically with respect to smoking policies; the companies' activities with respect to individual support measures for smokers remained low. Although dissemination was approached at a mass scale, it had modest reach and we observed only slight improvements in the advocated adoption of policies and programs. On the one hand, this might be explained by the societal context of this study: the dissemination activities probably could not generate any effect in* addition* to the historical trend. On the other hand, the fact that we did not observe clear benefits of the dissemination activities might also suggest the need for even more aggressive outreach and communication to target companies, or an acceptance that these channels do not seem to be sufficiently effective to merit investment. One might argue that legal regulations will be the adequate measure to ensure TP at worksites; however, as Radtke et al. [8] showed, a considerable proportion of the working population were still exposed to SHS at the worksite in 2010, even though the Swiss Federal law for protection against passive smoking was then in force. Therefore, further research is needed to explore more promising channels of dissemination in order to promote worksite TP. The finding of our study, indicating that improvements in TP measures at worksites are associated with improvements in aggregated health-related outcomes, shows that this will be worthwhile.

## Figures and Tables

**Table 1:** Changes in worksite TP between 2007 (*n* = 1627) and 2009 (*n* = 1452); cross-sectional data in percent (scale coding in brackets).

	There is no policy (0)	Smoking is allowed anywhere (1)	Smoking is allowed outside and in certain indoor areas (2)	Smoking is allowed outside, not in buildings (3)	Smoking is not allowed anywhere (4)
Prevalence and restrictiveness of smoking policies					
2007	5.8	4.1	47.6	40.0	2.5
2009	4.9	2.8	41.4	48.6	2.3

Number of individual support measures^*∗*^					
	0	1	2	3	

2007	85.0	11.6	2.8	0.6	—
2009	84.2	12.9	2.3	0.6	—

	Precontemplation (1)	Contemplation (2)	Preparation (3)	Action (4)	Maintenance (5)

Stage of change “smoke-free policy”					
2007	20.8	21.9	3.9	5.9	47.5
2009	14.5	15.8	3.7	5.5	60.5

Stage of change “cessation courses”					
2007	67.9	24.2	1.5	3.1	3.3
2009	66.9	22.1	1.8	5.7	3.5

^*∗*^For example, cessation courses, information material, or individual counseling for smokers.

**Table 2:** Longitudinal changes with regard to worksite TP and health-related outcomes aggregated at company level (Wilcoxon tests).

	Mdn_*t*1_^a^	Mdn_*t*2_^a^	Decrease	No change	Increase	*n*	*Z*	*p*	*r* ^b^
Worksite TP^1^									
Restrictiveness of smoking policy	2	3	9.4%	68.5%	22.1%	809	−5.941	<.001	−0.21
Number of individual support measures	0	0	9.1%	78.8%	12.1%	827	−1.609	.054	−0.06
SOC^2^ smoke-free policy	4	5	12.6%	54.8%	32.6%	786	−7.666	<.001	−0.27
SOC^2^ cessation courses	1	1	16.1%	65.6%	18.3%	771	−2.376	.009	−0.09

Health-related outcomes aggregated at company level									
% smokers in the workforce^3^	2	2	17.7%	56.0%	26.3%	723	−3.254	<.001	−0.12
SHS^4^-related problems^5^	1	1	31.4%	49.0%	19.6%	816	−6.195	<.001	−0.22
Absenteeism due to illness^5^	2	2	27.0%	46.3%	26.8%	800	−0.790	.215	−0.03

*Note.* ^a^Median.

^b^Effect size.

^1^For scale coding, see Table 1.

^2^Stage of change.

^3^1 = 0–20%, 5 = 80–100%.

^4^Environmental tobacco smoke.

^5^Answers were given on a five-point scale from 1 (“does not apply”) to 5 (“applies”).

**Table 3:** Differences between control group 2 (comprehensive brochure only) and control group 4 (monothematic brochure only) with respect to advances (*t*2 − *t*1 data) in worksite TP (*t*-tests).

	Control gr. 2		Control gr. 4		
	(*n* = 151^a^)		(*n* = 40^a^)		
	Mean	SD^b^	Mean	SD^b^	*t*
Differences^1^ in					
Restrictiveness of policy	.155	.8628	.244	.4889	−.632
Number of individual support measures	.055	.5325	−.023	.5112	.860
SOC^2^ smoke-free policy	.49	1.687	.45	2.062	.139
SOC^2^ cessation courses	.05	.889	.10	.810	−.345

*Note.* ^a^Companies who participated in both surveys and answered the respective questions both times.

^b^Standard deviation.

^1^ *t*2 data minus *t*1 data; for scale coding, see Table 1.

^2^Stage of change.

**Table 4:** Bivariate odds ratios for predictors (*t*1 data) of differences in worksite tobacco prevention (ordinal regressions).

	Differences (*t*2 minus *t*1 data) in			
	Policy restrictiveness^a^	Number of individual support measures^a^	SOC^b^ smoke-free policy^a^	SOC^b^ cessation course^a^
*Organizational attributes* ^1^				
Company size				
<50 employees	1.62	1.62	0.98	1.21
50–250 employees	1.82	1.90	0.97	0.98
>250 employees	ref.	ref.	ref.	ref.
Branch				
Construction	2.27^*∗∗*^	0.41^*∗∗*^	0.94	0.80
Hospitality	0.46^*∗∗*^	1.02	1.18	0.89
Health and welfare	0.83	0.91	0.77	1.58^*∗*^
Other	ref.	ref.	ref.	ref.
% women in workforce				
<20%	1.60^+^	0.77	0.83	0.92
20–39%	1.01	0.67	0.83	0.79
40–59%	1.16	0.81	1.14	0.79
60–79%	0.78	0.81	0.68	1.18
>80%	ref.	ref.	ref.	ref.
Other WHP^2^-measures	1.04	0.97	0.86^*∗*^	0.91

*Attributes of the representative* ^1^				
Personal concern	0.89^+^	0.90	0.80^*∗∗*^	0.88^+^
Perceived advantages^3^	0.79^*∗*^	0.94	0.59^*∗∗*^	0.94

*Dissemination strategies*				
Brochure only	0.97	0.53^*∗*^	0.87	0.56^*∗*^
Brochure and other^4^	ref.	ref	ref.	ref.
Number of contacts^5^	0.99	1.04	1.02	1.05
Type of intervention				
Monothematic intervention^6^	0.51	1.42	0.80	1.63
Comprehensive intervention^7^	ref.	ref.	ref.	ref.

*Note.* ^a^For scale coding, see Table 1.

^b^Stage of change.

^+^ *p* < .10; ^*∗*^ *p* < .05; ^*∗∗*^ *p* < .01.

^1^Measured at *t*1.

^2^Workplace health promotion (0 up to 6 measures, as stated in the questionnaire).

^3^Pros and cons for the respective measure, cons recoded.

^4^Information event, telephone marketing, and free initial consultation.

^5^In addition to brochure; as listed in the customer database.

^6^Control group 3 companies with intervention.

^7^Intervention group 2 companies with intervention.

**Table 5:** Bivariate odds ratios for predictors of differences (*t*2 data minus *t*1 data) in health-related outcomes aggregated at the company level (ordinal regressions).

	Differences (*t*2 minus *t*1 data) in		
	% Smokers	SHS^a^-related problems	Absenteeism
Change in policy restrictiveness			
Decrease	1.73^*∗*^	4.95^*∗∗*^	1.32
No change	0.91	2.91^*∗∗*^	1.21
Increase	ref.	ref.	ref.
Change in individual support measures			
Decrease	0.83	1.64^+^	1.14
No change	0.96	1.53^*∗*^	0.86
Increase	ref.	ref.	ref.
Change in SOC^1^ policy			
Decrease	1.77^*∗*^	2.47^*∗∗*^	1.53^*∗*^
No change	1.12	2.30^*∗∗*^	1.12
Increase	ref.	ref.	ref.
Change in SOC^1^ course			
Decrease	1.82^*∗*^	1.01	1.00
No change	1.12	0.99	0.98
Increase	ref.	ref.	ref.

*Note.* ^a^Environmental tobacco smoke.

^+^ *p* < .10; ^*∗*^ *p* < .05; ^*∗∗*^ *p* < .01.

^1^SOC = Stage of change.

**Table 6:** Sample composition of the baseline and follow-up studies.

	Baseline		Follow-up	
	(*n* = 1627)		(*n* = 1452)	
	*n*	%	*n*	%
Characteristics of the representatives				
Smoking status				
Nonsmoker	1198	74.0	1046	72.0
Occasional smoker	209	12.9	165	11.4
Smoker	212	13.1	184	12.7
Function				
CEO	820	49.7	703	48.4
Human resource manager	747	45.3	649	44.7
Health and safety manager	209	12.7	181	12.5
Authority to decide upon WHP measures				
Not authorised	762	47.6	741	51
Authorised	839	52.4	711	49

Characteristics of the organisations				
Size				
20–49 employees	1035	63.0	927	63.8
50–250 employees	528	32.2	435	30.0
>250 employees	79	4.8	76	5.2
Branch				
Construction	140	8.5	117	8.1
Hospitality	131	8.0	127	8.7
Healthcare and welfare	247	15.0	201	13.8
Other	1127	68.5	996	69.2
% Women				
<20%	472	29.6	414	28.5
20–39%	292	18.3	266	18.3
40–59%	414	25.9	370	25.5
60–79%	232	14.5	232	16.0
>80%	187	11.7	153	10.5

## Appendix

### A. Questionnaire Measures

The present research assessed the following variables at baseline and follow-up via a written questionnaire (Table 6).

*(1) Attributes of the Organisation.* The percentage of female employees was assessed by one item with given answer options in percent (see Table 4). To analyse the companies' previous practice with regard to workplace health promotion, we used four items to assess the extent to which health promotion measures (e.g., courses for general health behaviours) were already implemented at the workplace. Answers were given on a five-point scale (1 = not interested, 3 = intention to implement in the next months, and 5 = systematically implemented).

*(2) Attributes of the Representative.* Representatives indicated the extent to which they were personally concerned about tobacco prevention (e.g., “smoking is a private matter and none of the company's business”; 1 = disagree, 5 = agree) and how they evaluated a selection of possible pros and cons with regard to smoke-free workplaces and smoking cessation courses (health and economic benefits, rejection by employees and investments; 1 = disagree, 5 = agree).

* (3) Worksite Tobacco Prevention. *Participants used a single forced-choice item to indicate the extent to which tobacco prevention policies were already in place (0 = “there is no policy,” 1 = “smoking is allowed anywhere except a few nonsmoking areas,” 2 = “smoking is allowed outside and in certain indoor areas,” 3 = “smoking is allowed outside, but not in buildings,” and 4 = “smoking is not allowed anywhere”). To assess the prevalence and number (0–3) of individual support measures, we asked whether companies offered smoking cessation courses for their personnel, individual consultancy for smokers, or information material. To assess the stages of change with regard to worksite tobacco prevention, we used one item focussing on smoke-free policies and another focussing on cessation courses. Answers were given on a five-point scale with the following answer options: “We are not interested in adopting a smoke-free policy/cessation courses” (1, precontemplation); “We have not yet implemented a smoke-free policy/cessation courses but are interested in doing so” (2, contemplation); “We intend to implement a smoke-free policy/cessation courses in the next few months” (3, preparation); “Until now we have implemented a smoke-free policy/cessation courses only erratically” (4, action); “We have systematically implemented a smoke-free policy/cessation courses” (5, maintenance).

* (4) Health Outcomes*. The percentage of smokers was assessed by one item with given answer options in percent (1 =< 20%, 2 = 20–39%, 3 = 40–59%, 4 = 60–79%, and 5 => 80%). Problems related to environmental tobacco smoke (i.e., exposure to and complaints about environmental tobacco smoke) were assessed using a five-point rating scale (1 = disagree, 5 = agree). Absenteeism was assessed using a single item: “In our company we have a high level of absenteeism due to illness” (1 = disagree, 5 = agree).

Apart from the questions relating to previous practice and personal concern, all items included the response option “I don't know” in case an answer was not possible.
